# Crystal structure of thermostable alkylsulfatase SdsAP from *Pseudomonas sp. S9*

**DOI:** 10.1042/BSR20170001

**Published:** 2017-05-11

**Authors:** Lifang Sun, Pu Chen, Yintao Su, Zhixiong Cai, Lingwei Ruan, Xun Xu, Yunkun Wu

**Affiliations:** 1 State Key Laboratory of Structural Chemistry, Fujian Institute of Research on the Structure of Matter, Chinese Academy of Sciences, Fuzhou 350002, China; 2 Key Laboratory of Marine Biogenetic Resources, Third Institute of Oceanography, State Oceanic Administration (SOA), No. 178 Daxue Road, Xiamen 361005, China

**Keywords:** alkylsulfatase, crystal structure, Pseudomonas sp., SdsAP, SDS, SCP-2-like fold domain

## Abstract

A novel alkylsulfatase from bacterium *Pseudomonas sp. S9* (SdsAP) was identified as a thermostable alkylsulfatases (type III), which could hydrolyze the primary alkyl sulfate such as sodium dodecyl sulfate (SDS). Thus, it has a potential application of SDS biodegradation. The crystal structure of SdsAP has been solved to a resolution of 1.76 Å and reveals that SdsAP contains the characteristic metallo-β-lactamase-like fold domain, dimerization domain, and C-terminal sterol carrier protein type 2 (SCP-2)-like fold domain. Kinetic characterization of SdsAP to SDS by isothermal titration calorimetry (ITC) and enzymatic activity assays of constructed mutants demonstrate that Y246 and G263 are important residues for its preference for the hydrolysis of ‘primary alkyl’ chains, confirming that SdsAP is a primary alkylsulfatase.

## Introduction

Sulfatases are ubiquitous enzyme found in both prokaryotes and eukaryotes whose function is to catalyze the hydrolysis of the sulfate–ester bond, yielding the corresponding alcohol and inorganic sulfate [1,2,3]. To date, three mechanistically distinct types of sulfatases are identified: Cα-formylglycine-dependent sulfatases (type I) [4,5,6], sulfatase belonging to the Fe(II) α-ketoglutarate-dependent deoxygenate superfamily (type II) [7,2,8], and sulfatases belonging to the metallo-β-lactamase superfamily (type III) [9]. They play a key role in regulating the sulfation states of substrates [1,10,11]. Compared with eukaryotic sulfatases, which are involved in the desulfation of biomolecules to regulate cell signaling, hormone activity, and cellular degradation [6,1], prokaryotic sulfatases are primarily involved in assimilating sulfur or utilizing alkyl- and aryl-sulfonates as a carbon and/or sulfur source for cell growth [12,13]. Such as SdsA1 (type III) from *Pseudomonas aeruginosa* that enables the bacterium to utilize the sodium dodecyl sulfate (SDS) as a sole carbon source to survive [9,14].

In recent years, SDS has extensively been used in industries and daily life because of its favorable physicochemical properties [15]. Due to this, the biodegradation of SDS from the environment and avoidance of secondary pollution have gained much importance [16]. However, few sulfatases to date have been used widely for the application of SDS biodegradation.

**Figure 1:**
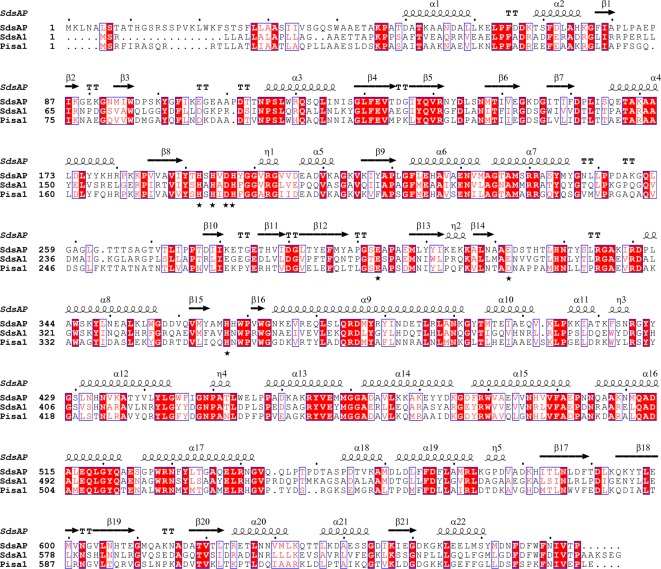
Structure-based sequence alignment of SdsAP (F2WP51), Pisa1 (F8KAY7), and SdsA1 (Q9I5I9). Identical residues are shown on red background and similar residues are underlined by blue boxes. Some residues involved in active site and Zn ion coordination is marked by asterisk. The figure was generated by ClustalW and ESPript.

The detailed structural interpretations of sulfatases provide valuable information for addressing their catalytic mechanisms. Till now, the three-dimensional structures of type III alkylsulfatases, SdsA1 and Pisa1, have been characterized and interpreted [9,17]. They show a high structural similarity and a slight difference in their active site region. As described, they share a distinct metallo-β-lactamase fold domain, a dimerization domain and a sterol carrier protein type 2 (SCP-2)-like fold domain. It has been suggested that SdsA1 is a primary alkylsulfatase preferring the hydrolysis of ‘primary alkyl’, while Pisa1 is a secondary alkylsulfatase preferring the hydrolysis of ‘secondary alkyl’ sulfates[9,17]. Recently, a novel sulfatase, SdsAP, was identified from a newly isolated bacterium *Pseudomonas sp. S9* and enzymatic assays proved its ability to hydrolyze the primary sulfates like SDS [18]. SdsAP shares 42% and 46% sequence identity with SdsA1 and Pisa1 respectively (Figure 1). Interestingly, SdsAP was reported as a thermostable enzyme that had an optimal activity at 70°C and still kept more than 90% activity after treatment at 65°C for 1 h [18]. Therefore, SdsAP is an ideal candidate for the application on the degradation of SDS-containing waste.

Here, we present the crystal structure of SdsAP from *Pseudomonas sp. S9* at 1.76 Å. The structural comparison and well superimposability of the active site region between SdsAP and SdsA1 imply that SdsAP is a primary type-III alkylsulfatase. Mutations of residues Tyr246 and Gly263 of SdsAP show that the mutants abolish the enzyme activity for SDS degradation, indicating these residues are important to its substrate preference.

## Materials and methods

### Protein expression and purification

The amplified SdsAP gene from the chromosomal DNA of *Pseudomonas sp. S9* was cloned into vector pET-His for expression. Its N-terminal signal peptide of 41 amino acids was truncated. The recombinant protein was expressed at 37°C in *Escherichia coli* strain BL21 (DE3) cells and induced with 0.3 mM IPTG at 16°C for 15 h in LB media at an OD_600_ of 0.6–0.8. Cells were harvested by centrifugation and then resuspended in lysis buffer containing 50 mM Tris/HCl (pH 8.0), 300 mM NaCl, 5% glycerol, and sonicated on ice. Recombinant protein was purified from the supernatant by IMAC column (GE Healthcare) and digested with thrombin (Sigma) overnight at 4°C, followed by ion exchange chromatography on Mono Q and gel filtration chromatography on a Superdex 200 column (GE Healthcare). Finally, the purified protein was concentrated to 27.5 mg/ml by ultrafiltration in 25 mM Tris/HCl (pH 8.0), 200 mM NaCl, and 5% glycerol.

### Site-directed mutagenesis

The SdsAP mutants were prepared according to the protocol described in QuikChange Site-Directed Mutagenesis Kit [19]. Plasmid pET-His-SdsAP was used as the template for the introduction of desired mutations. The mutations were introduced by PCR using the appropriate primers listed in Table 1. After PCR, the amplified plasmids were digested for 1 h at 37°C with DpnI and then transformed into *E. coli* DH5α. Mutants Y246A, Y246S, G263A, G263F, Y246A/G263A, and Y246S/G263F were confirmed by DNA sequencing. Pisa1 plasmid was provided by Prof. Kurt Faber from University of Graz, Austria. The SdsAP mutants and Pisa1 were expressed and purified as described above for SdsAP.

**Table 1:** Oligonucleotide primers used in the present study

Primers	Primers sequence (5′–3′)
Y246A-F:	GGCCAGCTATATGGCCGGTAACCTGCTGC
Y246A-R:	GCAGCAGGTTACCGGCCATATAGCTGGCC
Y246S-F:	GGCCAGCTATATGAGCGGTAACCTGCTGC
Y246S-R:	GCAGCAGGTTACCGCTCATATAGCTGGCC
G263A-F:	TAGGCGCTGGTCTGGCAACCACCACATCGG
G263A-R:	CCGATGTGGTGGTTGCCAGACCAGCGCCTA
G263F-F:	TAGGCGCTGGTCTGTTCACCACCACATCGG
G263F-R:	CCGATGTGGTGGTGAACAGACCAGCGCCTA

### Isothermal titration calorimetry

Enzyme rate assay was carried out by an ITC200 calorimeter (GE Healthcare) on SdsAP hydrolase using the SDS substrate, according to the manufacturer’s instructions (Method 2A: Enzyme assay–substrate only). All experiments were performed at 25°C in 25 mM Tris/HCl (pH 8.0), 200 mM NaCl buffer. Initially, the enthalpy of the reaction was determined by the multiple injection method. The substrate SDS (3 mM) was prepared in the buffer and placed in syringe for titrating (2 μl × 12, duration of 30 s, spacing time of 900 s) to enzyme (2 μM). The rate of heat generated (power: d*Q*/d*t*) at each substrate concentration is carried by the titration of enzyme (25 nM) with SDS (3 mM, 2 μl × 20, duration of 4 s, spacing time of 180 s). The Michaelis–Menten fit was obtained by Model2 Substrate Only fitting with ORIGIN version 7.5 (MicroCal).

### Enzyme activity assays for SdsAP and its variants

Enzyme activities of SdsAP and its variants for the hydrolysis of SDS were analyzed using stains-all solution method [18,20] by an UV-1100 spectrophotometer equipped (MAPADA). Fifty microliters of diluted enzyme solution (0.15 mg/ml) was mixed with 450 μl of 25 mM Tris/HCl (pH 7.1), 200 mM NaCl, and 5% glycerol containing 50 μg of SDS. The final concentration of enzyme and SDS in the assay is 0.015 mg/ml and 0.1 μg/μl respectively. After incubation at room temperature for 10 min, the reaction was terminated by adding 20 μl of sample solution to 980 μl of stains-all solution and then measured at 438 nm. The SDS quantitation was measured at its maximum absorbance of 438 nm and compared with the standard curve of SDS.

### Crystallization, structure determination, and refinement

Crystals were obtained by mixing 1 μl of SdsAP protein (27.5 mg/ml) with 1 μl of reservoir solution composed of 0.1 M sodium acetate (pH 4.5), 0.05 M magnesium acetate, 20% v/w polyethylene glycol 4000, and submitting to sitting drop vapor diffusion at 293 K. The crystals of SdsAP were cryoprotected by immersion in reservoir solution supplemented with 25% glycerol followed by transferring to liquid nitrogen, and then maintained at 100 K during X-ray diffraction data collection using the beamline BL17U at Shanghai Synchrotron Radiation Facility (SSRF, Shanghai, China) [21]. The data were processed by using HKL2000 [22] and CCP4 suites [23].

The crystal structure of SdsAP was determined by the molecular replacement, using the SdsA1 structure (PDB: 2CG3) as the search model in PHASER [24]. After generation of the initial model, iterative cycles of manual rebuilding using Coot [25], and maximum likelihood refinement with PHENIX were performed [26]. The figures of the structures were prepared by using PyMOL program (DeLano Scientific LLC). The solvent accessible surface area and buried surface area were calculated using CCP4 suite [23,27]. The atomic coordination and structure factors for the SdsAP have been deposited in the Protein Data Bank under the accession code of 4NUR.

## Results and discussion

### Structure of SdsAP

**Figure 2:**
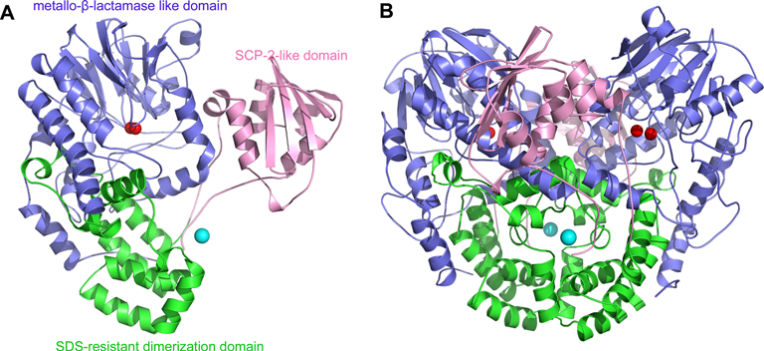
The structure of SdsAP (**A**) A cartoon representation of the SdsAP. The N-terminal metallo-β-lactamase-like domain was colored by slate; SDS-resistant dimerization domain was colored by green; the C-terminal SCP-2-like domain was colored by pink; Zn ion was colored by red; Mg ion was colored by cyan. (**B**) The dimer of SdsAP.

The final SdsAP was refined to a resolution of 1.76 Å with *R*_work_ of 15.22% and *R*_free_ of 18.40%. The statistics of data collection and refinement statistics are presented in Table 2. In the crystal structure, each SdsAP monomer has a featured type-III-sulfatase fold, consisting of the N-terminal metallo-β-lactamase like domain (residues 42–401, a 14-stranded β-sandwich surrounded by α-helices, αββα-sandwich domain, blue), SDS-resistant dimerization domain (402–543, an α-helical domain, green), and the C-terminal SCP-2-like domain (544–674, a five-stranded β-sheet core and six helical, pink) (Figure 2). In an asymmetric unit, two SdsAP monomers are related by a non-crystallographic 2-fold axis and form a large dimer interface including residues from all the three domains. The dimer interface has a buried surface area of approximately 9818 Å^2^ and account for 21.5% of total surface area of SdsAP monomer.

**Table 2:** Data collection and refinement statistics

**Data collection**	
Space group	P 2_1_
Cell dimensions	
a, b, c (Å)	89.78, 76.66, 103.18
α, β, γ (°)	90, 95.03, 90
Resolution (Å)	1.76
*R*_merge_ (%)	10.5 (93.5)
I/I_δ_	16.41 (2.54)
Completeness (%)	99.90 (99.90)
Redundancy	4.2 (4.0)
Wilson B-factor (Å^2^)	18.21
**Refinement**	
Resolution (Å)	1.76–39.75
Number of reflections	137185
*R*_work_/*R*_free_ (%)	16.10/19.10
Number of atoms	
Protein	9963
Zn	4
Mg	2
Water	1423
R.m.s.d bonds (Å)	0.006
R.m.s.d angles (°)	0.823
Ramachandran plot	
Favored (%)	97.46
Allowed (%)	2.46
Outliers (%)	0.08
Rotamer outliers (%)	0.00

Numbers in parentheses refer to the highest resolution shell.

### Structure comparison

**Figure 3:**
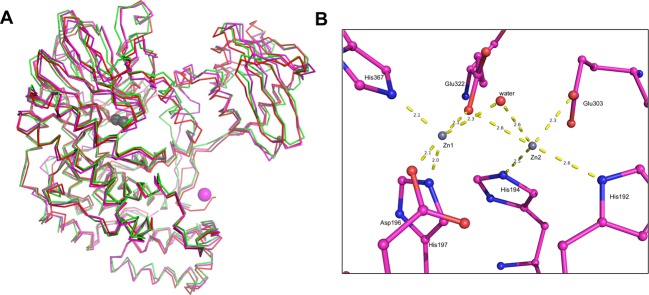
Structure alignment (**A**) Superimposed crystal structures of SdsAP (magenta), SdsA1 (2CFU, slate, 2CG3, red), and Pisa1 (4AXH, green) as a ribbon diagram. (**B**) The Zn^2+^-binding site. Zn ions are depicted as gray spheres, water molecules or hydroxyl ions as red spheres, and side chains coordinating Zn ions were labeled and presented as thicker sticks. Mg ion is depicted as magenta sphere.

The search of the PDB database for structurally similar protein using DALI server indicates that SdsAP shares high structural similarity with SdsA1 and Pisa1. The high homology at the tertiary structural level is further manifested in the structure overlay with the well-superimposed regular secondary structure elements among SdsAP, SdsA1, and Pisa1 (Figure 3A). Superposition of backbone of SdsA1 (PDB: 2CG2) and Pisa1 (PDB: 4AXH) with SdsAP shows Cα RMSD values of 1.4 Å and 1.4 Å respectively (Figure 3A).

**Figure 4:**
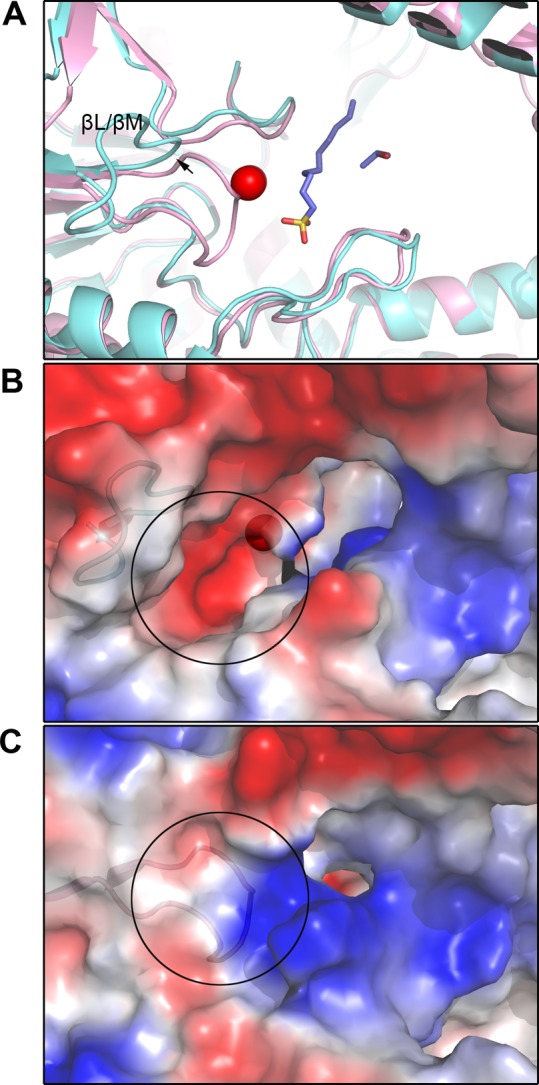
The conformation changed of the βL/βM loop (**A**) Cartoon comparison of the βL/βM loop of SdsAP (pink, closed) and SdsA1 (2CFU, cyan, open). Sulfate ion and 1DA were modeled and shown by sticks. (**B**) The electrostatic potential surface of corresponding βL/βM loop in SdsA1 and SdsAP. (**C**) Blue is for positive charge whereas red is for negative charge.

Comparison of the zinc-binding sites of the three alkylsulfatases SdsA1, Pisa1, and SdsAP reveals a nice conservativity, despite the different substrate specificities and regiospecificities. Similar to SdsA1, zinc ions locate at the internal edge of the two central β-sheets, suggesting the active site. One zinc ion has a trigonal pyramidal coordination sphere where His^197^, His^367^, and one water molecule provide the equatorial and Asp^196^ and Glu^322^ the apical ligands (Figure 3B), equivalent Zn1 in SdsA1 [9]. Zn2 of SdsA1 is tetrahedrally coordinated by His^192^, His^194^, Glu^303^, and Glu^322^, equivalent Zn2 in SdsA1. The distances of Zn1 coordinating atoms are closer than that of Zn2. Thus, Zn2 is lost more easily, which is in agreement with the previous reports [9,18]. Similar to SdsA1, βL/βM loop is present at a closed conformation with charged residues embeded inside (Figure 4A and C), while the charged residues are exposed outside and Zn2 is lost in its open conformation (Figure 4B). The two conformations of βL/βM loop may be important to the entrance of the metal ion necessary for enzymatic activity.

The metallo-β-lactamase-like domain of SdsAP holds the conserved bucket shape architecture with an internal active cavity in accordance with most metallo-β-lactamase [28]. All of them share a similar αββα-fold and possess two potential zinc ion-binding sites. In the case of B1 enzymes (BcII, CcrA), one zinc ion possesses a tetrahedral coordination sphere and is coordinated by His^116^, His^118^, His^196^, and a water molecule or OH^−^ ion, which named the ‘histidine’ site [29,30]. The other zinc ion has a trigonal pyramidal coordination sphere that involves Asp^120^, Cys^221^, His^263^, and two water molecules that named ‘cysteine’ site. In SdsAP or SdsA1, the His^196^ is replaced by a glutamate in the ‘histidine’ site, while in Pisa1, it is replaced by an aspartic acid. In the ‘cysteine’ site, the Cys^221^ is replaced by a glutamate in SdsAP, SdsA1, and Pisa1. So far, the glutamate in the direct vicinity of the zinc ions is unique to these three alkylsulfatases.

**Figure 5:**
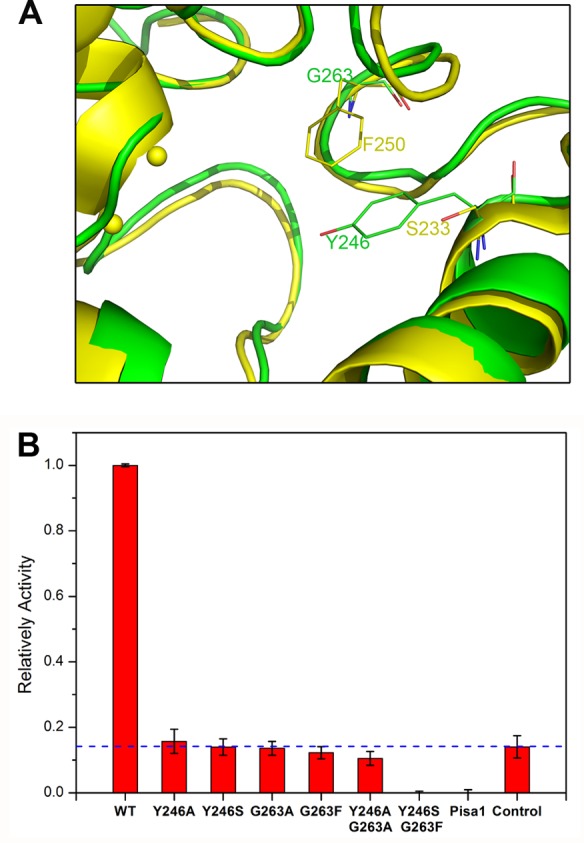
Enzymatic activities analysis (**A**) Comparison of the active sites between SdsAP (Green) and Pisa1 (Yellow). (**B**) Enzymatic activities analysis of SdsAP and its variants with SDS as a substrate. The control is estimated without enzyme. Three parallel experiments were performed in the assay. Relatively activity’s value of control is indicated with dash line. Values of relatively activity lower than control are considered to no enzyme activity.

The substrate-binding sites of SdsAP and SdsA1 are essentially superimposable, suggesting that two sulfatases share the similar catalytic mechanism. As reported in Pisa1, Ser^233^ and Phe^250^ are important residues to its preference for shorter alkyl chains, leading Pisa1 as a secondary alkylsulfatase [17]. However, in the equivalent position, there are Tyr^246^ and Gly^263^ in SdsAP, while Tyr^223^ and Gly^240^ in SdsA1 (Figure 5B). Therefore, considering the higher structural conservativity of SdsA1 and SdsAP at the active site, it indicates both of them could be a primary alkylsulfatase.

### Enzyme activity assay

To study the role of Tyr^246^ and Gly^263^ in SdsAP’s substrate preference, we generated several single and double mutations by active site-directed mutagenesis: Tyr246Ala, Tyr246Ser, Gly263Ala, Gly263Phe, as well as double mutations Tyr246Ala/Gly263Ala and Tyr246Ser/Gly263Phe. In the enzymatic activity assays, SDS was used as a substrate and the varied quantity could be obtained by measuring SDS’s maximum absorbance at 438 nm. Compared with the relatively activity of wild-type SdsAP, not only the single Tyr^246^ mutations but also Gly^263^ mutations had a very small value, suggesting the enzymatic activities were abolished for both (Figure 5). Furthermore, the double mutation Tyr246Ser/Gly263Phe of SdsAP could not restore enzyme activity and hydrolyze the SDS, as observed for Pisa1.

**Figure 6:**
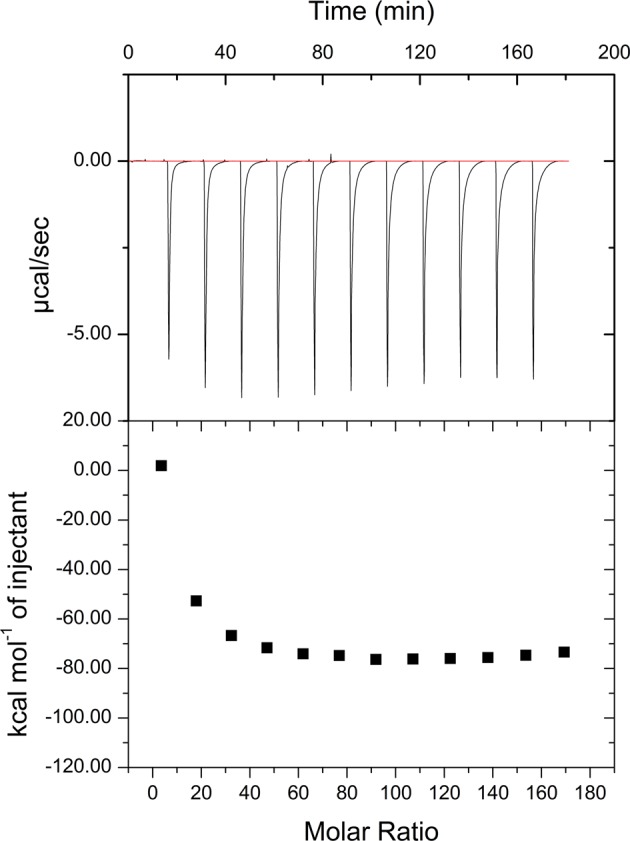
Determination of ΔH_app_ for the hydrolysis of 3 mM SDS by 2 μM SdsAP at 25°C. The mean ΔH_app_ for ten injection is a value of -74.74 kcal/mol.

**Figure 7:**
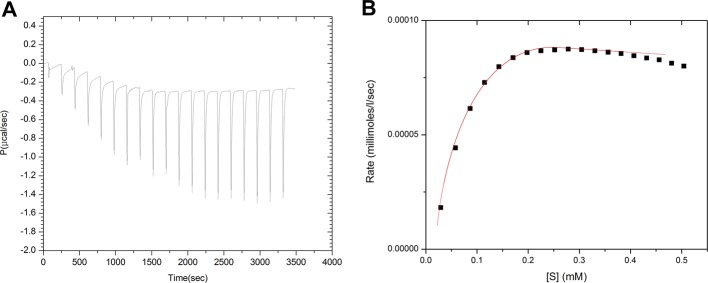
Determination of the kinetic parameters by ITC. (**A**) Raw calorimetric data and (**B**) calorimetric determination of enzyme kinetic parameters for the hydrolysis of 3 mM SDS by 25 nM SdsAP at 25°C.

Kinetic characterization of SdsAP was performed by isothermal titration calorimetry (ITC) with SDS as a substrate. Initially, by using a multiply injection methodology to determine the apparent molar enthalpy (ΔH_app_) (Figure 6), the mean ΔH_app_ for ten injections is initially measured with a value of −74.74 kcal/mol. And then the rate of enzymatic substrate turnover as a function of substrate concentration was measured (Figure 7). These data were fitted to the Michaelis–Menten equation with single binding site model and the calculated kinetic parameters for SdsAP were *K*_m_ = 74.2 ± 10 μM and *K*_cat_ = 4.88 ± 0.17 s^−1^. However, neither of these variants had measureable activity to SDS. Thus, the results suggest that both Tyr^246^ and Gly^263^ play a crucial role in enzyme activity of SdsAP, and would be the key residues for its substrate preference for primary alkyl chains.

In summary, we present a high resolution structure of SdsAP, a thermostable alkylsulfatase from *Pseudomonas sp. S9*. The overall three-dimensional structure is a symmetric dimer with a large dimer interface. Each monomer of SdsAP is characterized by the typical type-III-sulfatase globular fold, showing a high structural similarity to SdsA1 and Pisa1. In comparison of their active site residues, distinct difference is pinpointed. Furthermore, site-directed mutagenesis assays indicates that both Tyr^246^ and Gly^263^ of SdsAP are crucial residues for its substrate preference, confirming that SdsAP is a member of the primary alkysulfatases and should be an ideal enzyme with high thermostability to degrade the SDS-containing waste. Therefore, our structural and functional studies of SdsAP will provide a basis for further enzymatic modification and potential application.

## Abbreviations

ITC: isothermal titration calorimetry
SCP-2: sterol carrier protein type 2
SDS: sodium dodecyl sulfate
CCP4: collaborative computational project number 4
